# Effects of a Worksite-Based Self-Management Program in Traditional Retail Market Workers: Longitudinal Associations with Metabolic Parameters

**DOI:** 10.3390/ijerph19052854

**Published:** 2022-03-01

**Authors:** Yeon-Ha Kim, Seong-Yong Yoon

**Affiliations:** 1 Department of Nursing, Korea National University of Transportation, Chungbuk 27909, Korea; tiffany7@ut.ac.kr; 2 Division of Occupational and Environmental Medicine, School of Medicine, Soonchunhyang University Gumi Hospital, Gyeongbuk 39371, Korea

**Keywords:** metabolic syndrome, self-management, small business, community-based participatory research, checklist

## Abstract

(1) Background: This study explored the effects of a worksite-based self-management program on metabolic parameters in traditional retail market workers during a 3-year period. (2) Methods: Seventy traditional retail market workers who completed 3 years of follow-up were included in this study. The worksite-based self-management program was developed to help traditional retail market workers manage metabolic parameters themselves based on the following content: (I) using a metabolic syndrome action checklist, (II) counseling, (III) creating improvement action plans, and (IV) evaluating implemented improvements. (3) Results: The rates of implemented improvements showed success. Fasting blood sugar and triglycerides showed yearly reductions from baseline for 3 years, which eventually led to a decreased metabolic syndrome score and maintenance of metabolic parameters in the normal range for 3 years. (4) Conclusions: A worksite-based self-management program on metabolic parameters in traditional retail market workers was effective. It improved their intention to self-assess and cope their health problems and eventually decreased their metabolic syndrome score. It should be considered for the primary prevention of metabolic syndrome among traditional retail market workers.

## 1. Introduction

Metabolic syndrome (MetS) is defined as a cluster of risk factors and is regarded as a target for preventing early stages of coronary heart disease [1,2]. According to the Korea National Health and Nutrition Examination Survey, the age-standardized prevalence of MetS in the Korean population is 20.3% [3]. It has been reported that workers with more irregular lifestyles—defined in terms of factors such as sleep duration, frequency of breakfast, working hours, and work type—have a higher prevalence of MetS [4]. As MetS is known to be more prevalent in middle-aged individuals, improvements in lifestyle habits focusing on physical activity, reduction of alcohol intake and smoking, dietary intake, and coping with stress can normalize metabolic disorders [5,6].

Workers of traditional retail markets, which are typical small businesses that provide retail services, are known to have a higher risk of MetS than workers of large enterprises [7]. It has been reported that 74.8% of workers from small enterprises and retail services are over 50 years old and that 81.3% of them work more than 10 h per day, with work hours ranging from 5 AM to 10 PM, while having a very low income [8,9]. The average store area is 30.4 m^2^, which likely causes a high stress level and musculoskeletal symptoms [8,9]. Habits such as skipping meals or eating fast food, frequent alcohol intake, smoking, and low physical activity have been reported [10]. These conditions impose an adverse effect on health that can act as a barrier to sustainable health behavior. It has been reported that 56.9% of these workers have abnormal values for one or two metabolic parameters [10]; nevertheless, traditional retail market workers usually provide face-to-face services, making it difficult to leave their stores and participate in health education programs or health screening. As a result, they are likely to not engage in proper health management. Thus, a self-managed long-term intervention program needs to be promoted for traditional retail market workers so that they can better manage their health behaviors, voluntary improve their lifestyles, and implement sustainable improvement actions for meaningful and practicable changes [11,12].

Although implemented actions proposed for health behavioral outcomes are good indicators of whether a self-management program can improve health behavior [13], most self-management programs have only measured clinical outcomes. They have rarely assessed implemented actions because of uncertainty regarding which health problems to target and which actions to improve [14]. Thus, an action checklist needs to be developed so that workers can design and implement improvement actions for their health conditions more easily [12,15]. Moreover, it should be easy to evaluate the implementation of actions proposed to improve health behavioral outcomes. Action checklists have been widely developed for workers of small to medium enterprises, agricultural facilities, construction sites, and informal economy workplaces. They have also been applied in participatory intervention programs for work-related musculoskeletal diseases [16,17], mental health [18], needle sticks [19], MetS [20,21], and cardiovascular disease [22]. However, no action checklist tailored to modify metabolic parameters for traditional retail market workers has been developed or applied in a self-managed intervention program.

Therefore, the objective of this study was to explore the sustainable effects of a worksite-based self-management (WBSM) program on metabolic parameters in traditional retail market workers during a 3-year period.

## 2. Materials and Methods

### 2.1. Participants and Procedures

This was a prospective follow-up study of participants during 3 consecutive years. A WBSM program to improve metabolic parameters was conducted in four traditional retail markets located in Gumi, North Gyeongsang Province, South Korea. Among all workers in four traditional retail markets, subjects were selected based on the following inclusion criteria: (1) those who had worked in those markets for more than 2 years; (2) those who were over 40 years of age; (3) those who were considered to be at risk of MetS based on results of regular health screening examinations if three or more abnormalities were present when using criteria of National Cholesterol Education Program Expert Panel (NCEP EP) III [23]; and (4) those who were in the middle- or high-risk for cardiovascular disease according to the KOSHA guidelines for the assessment and management of cardiovascular disease [24]. The exclusion criteria were: (1) those who were diagnosed with a severe psychiatric disorder or cognitive impairment; (2) those who were diagnosed with musculoskeletal, neurological vascular, lung, or cardiac problems; (3) those who were pregnant; and (4) those who were unable to participate in this study.

**Figure 1:**
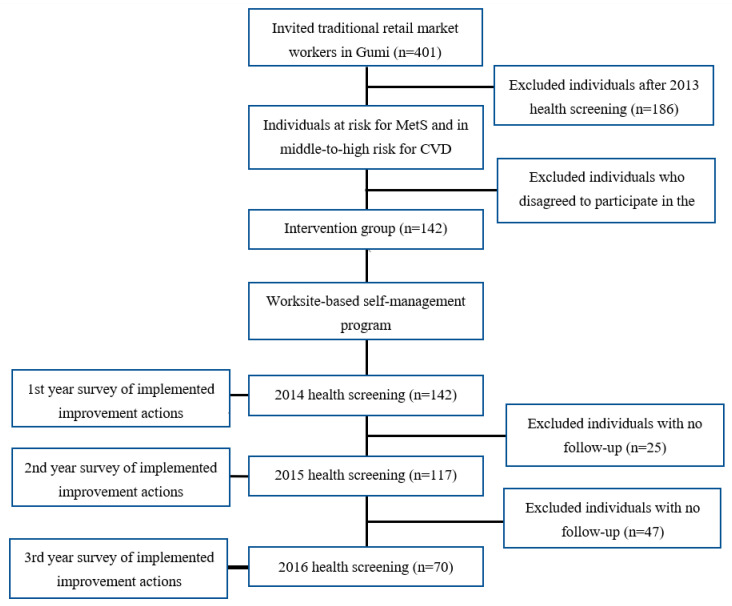
Flowchart showing enrollment of participants. MetS = Metabolic Syndrome; CVD; Cardiovascular Disease.

This study was conducted from March 2013 to December 2016. The Gumi public health center invited 401 traditional retail market workers for health screening examinations. After excluding those who did not have a risk factor for MetS and had a low risk of cardiovascular disease (*n* = 186) and those who did not agree to participate in this study after a detailed explanation of the program (*n* = 73), a total of 142 subjects were enrolled in this study (2013). Baseline surveys were conducted using annual health screening results (2013) and after the WBSM program was applied, participants continued to carry out WBSM by themselves. As post-trial follow-up data, annual health screening results from 2014 (*n* = 142), 2015 (*n* = 117), and 2016 (*n* = 70) were used, and implemented actions from the action plans were also assessed after 3 months and 6 months, respectively, for 3 years. In the current study, 70 participants who completed health screening examinations in 2013, 2014, 2015, and 2016 were included to explore the sustainable effectiveness of the WBSM program (Figure 1). The main reason for dropout was that despite repeated calls to encourage participants to participate in follow-up, they did not attend the health screening examination (Figure 1).

### 2.2. Development of an Intervention Program

A specifically tailored WBSM program targeting metabolic parameters for traditional retail market workers was designed based on the principles of participatory action-oriented training (PAOT) programs, which include building upon local practice, focusing on achievements, using a learning-by-doing approach, encouraging exchanges of experience, and promoting people’s involvement [12,15]. Using these concepts as a theoretical framework, the WBSM program was developed to help traditional retail market workers to manage their metabolic parameters based on the following content: (1) using a MetS action checklist, (2) counseling, (3) creating improvement action plans, and (4) evaluating implemented improvements. Participants were asked to use the MetS action checklist to identify actions for improvement and propose practical health improvements applicable in a range of specific traditional retail market worksites [15]. Counseling was delivered to help participants recognize solutions through self-reliance and plan practical actions [25]. At the end of the third month and the sixth month, respectively, counselors supported and evaluated implemented improvement actions [12].

#### 2.2.1. Development of the Action Checklist

The MetS action checklist was developed by modifying the action checklist for cardiovascular disease developed by Yoon et al. [22]. Main areas and several checkpoints were modified to improve the health behaviors of traditional retail market workers for dealing with the risk of MetS. The original action checklist consisted of nine main areas and 62 checkpoints. The MetS action checklist consisted of eight main areas with 62 checkpoints. These eight main areas were as follows: hypertension management, dyslipidemia management, diabetes management, alcohol drinking management, smoking habits management, exercise management, diet management, and stress management. The checkpoints consisted of practical health solutions for traditional retail market workers to manage and prevent the risk of MetS built on effective practice. Content validity was verified by three occupational health medicine professors and three community nursing professors who had experience with PAOT and participated as facilitators (Table 1).

#### 2.2.2. Counseling and Setting Action Plans

Individual counseling was provided by nurses from a community health center for 30 min. Three experienced trainers (a nurse from a community health center and two community health workers) visited individual participants while they were at work. The two community health workers handled participants’ work responsibilities while the nurse delivered counseling. First, participants assessed their own health conditions regarding MetS using the MetS action checklist. Then they identified points to maintain and points to improve. In addition, they proposed priority actions as a plan for improving their health behaviors to deal with MetS [15]. The counselor praised participants if there were well-practiced points and explained to participants if there were significant points to be improved by sharing good examples of experiences of other traditional retail market workers with a similar working environment with a similar risk of MetS. Through this process, participants were encouraged to find practicable solutions for improving their existing health conditions and to set action plans that they could implement immediately at their workplace to change their health behaviors. Each participant was required to record their action plans on a commitment sheet. After the first counseling session, follow-up counseling was given at 3 and 6 months, respectively, to evaluate participants’ achievement of the implemented action plans based on their self-monitored records. Participants were praised and acknowledged if they had implemented actions and encouraged to find more effective ways of doing so if there were unpracticed plans. Their questions were also answered. Each follow-up counseling lasted for 20 min.

After the program was applied, participants spontaneously carried out worksite self-management actions based on the content of the WBSM program every year by using the MetS action checklist to create action plans and implement improvement actions. The follow-up visit was given to encourage participants and to survey implemented actions based on self-evaluations after 3 and 6 months.

### 2.3. Measurements

The outcome measurments were changes in the implementation of action plans and changes in metabolic parameters. The implementation rate of action plans was surveyed every 3 months and 6 months. Metabolic parameters such as waist circumference (WC), systolic blood pressure (SBP), diastolic blood pressure (DBP), fasting blood sugar (FBS), triglycerides (TGL), and high-density lipoprotein cholesterol (HDL-C) were checked using annual health screening data for all participants as baseline and a post-trial follow-up test. Metabolic parameters were considered by NCEP EP III definition to be abnormal if they met the following criteria: WC ≥ 90 cm for men or ≥85 cm for women, SBP ≥ 130 mmHg and/or DBP ≥ 85 mmHg and/or FBS ≥ 100 mg/dL and/or TGL ≥ 150 mg/dL and/or HDL-C < 40 mg/dL [23]. A self-administered questionnaire with items on gender, age, working time, smoking, drinking alcohol, stress, and occupation-related musculoskeletal pain was given before the WBSM program. Working hours per day and alcohol consumption days per week were assessed. Smoking, stress, and occupation-related musculoskeletal pain status were assessed using a dichotomous scale (yes or no).

### 2.4. Data Analysis

All statistical analyses were performed using SPSS version 23.0 (IBM Corp., Armonk, NY, USA). Means (standard deviation) and frequencies (%) were used to analyze descriptive data. Differences in the program’s effect from baseline until follow-up in 2014, 2015, and 2016 were examined using repeated-measures analysis of variance for the normally distributed WC variable. For the non-normally distributed variables (SBP, DBP, FBS, TGL, HDL-C, and MetS score), the Wilcoxon test and Friedman test were performed. *p* values <0.05 were considered to indicate statistical significance.

### 2.5. Ethical Considerations

The researchers received ethical approval for this study. Institutional Review Board (IRB) approval was obtained from the researchers’ affiliated university hospital (IRB No. SCHUH Medicine 2020-18). Each participant was informed of the purpose of this study, their right to withdraw without penalty, and assurance of the confidentiality and anonymity before providing written informed consent.

## 3. Results

### 3.1. General Characteristics

Table 2 shows the characteristics of the study subjects. Among 70 subjects, 49 (69.8%) were women. The majority (82.9%) of subjects were more than 50 years old. Their mean age was 58.15 ± 8.51 years. Most (94.9%) subjects worked for more than 10 h a day. Out of all the subjects, 32.5% were smokers, 32.8% drank alcohol more than one day per week, 87.2% were exposed to stress, and 54.2% had a musculoskeletal disease.

### 3.2. Proposed and Implemented Action Plans during 3 Years

Table 3 shows the number of proposed and implemented MetS action plans during the 3-year study period. In the first year, the total number of action plans in every action checklist area was 296. The success rate of implemented improvements was 82.7% after 3 months and 61.4% after 6 months. In the second year, 281 actions were planned and 86.8% and 57.6% of them were implemented after 3 months and 6 months, respectively. In the third year, 246 actions were planned and 77.2% and 53.2% of them were implemented after 3 months and 6 months, respectively.

### 3.3. Follow-Up Results of MetS Risk Components during 3 Years of Follow-Up

**Figure 2:**
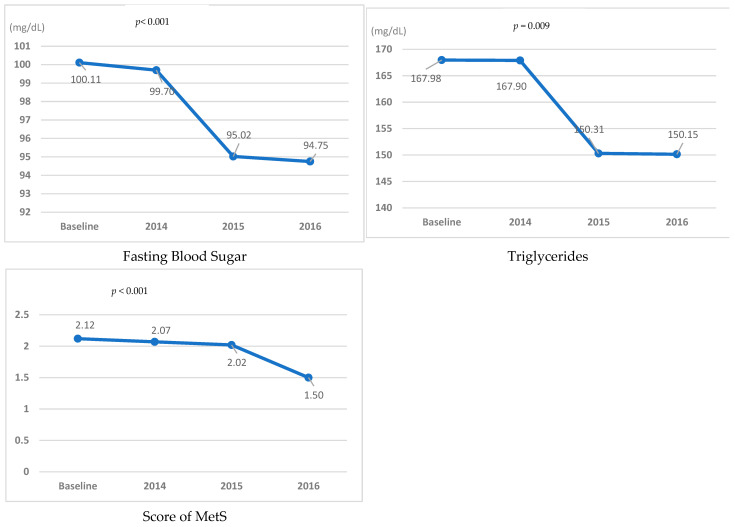

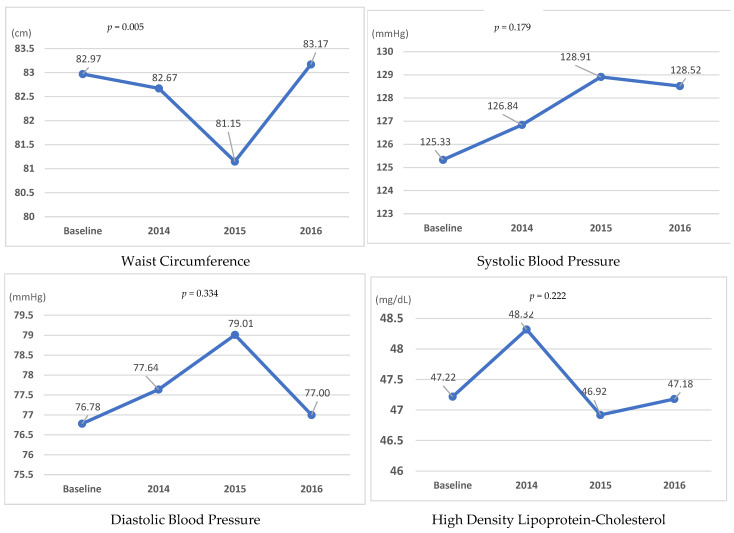
Metabolic syndrome (MetS) risk components compared to the baseline.

Table 4 presents follow-up results regarding MetS risk components for 3 years. FBS (χ^2^ = 18.52, *p* < 0.001), TGL (χ^2^ = 11.68, *p* = 0.009) and MetS score (χ^2^ = 17.73, *p* < 0.001) showed significant differences. WC significantly decreased in 2015 (*p* = 0.009) compared to baseline, but it increased nonsignificantly in 2016 (*p* = 0.753). FBS (*p* = 0.005) and TGL (*p* = 0.009) significantly decreased in 2016 compared to baseline. TGL significantly decreased in the third year (*p* = 0.009) compared to baseline. Although the MetS score remained in the normal range, it significantly decreased in 2016 (*p* < 0.001) compared to baseline (Figure 2).

## 4. Discussion

This study developed and applied a WBSM program targeting metabolic parameters for traditional retail market workers with 3 years of follow-up to determine its sustained effects on metabolic parameters. Various intervention methods were applied in this program, including the development of a MetS action checklist, counseling, creating action plans for improvement, and evaluating implemented improvements to highlight the strengths and supplement the weaknesses of each applied method [12]. To the best of our knowledge, no previous studies have estimated the effectiveness of an intervention program for MetS among traditional retail market workers, especially for promoting their self-management. Therefore, we cannot compare our results directly to those of previous research.

The results of this study showed that the self-assessment of health conditions regarding MetS-related improvement actions produced practicable changes useful for traditional retail market workers. In our study, the MetS action checklist was designed for use as a tool for setting goals. We observed that 82.7%, 86.8%, and 77.2% of proposed action plans were implemented within 3 months after the WBSM program in the first, second, and third years, respectively. The compliance with the action plan showed continuance, with compliance rates of 61.4%, 57.6%, and 53.2% within 6 months after the WBSM program in first, second, and third year, respectively. This coincided with previous studies demonstrating that compliance with an action plan was maintained for periods of 3 and 6 months after an intervention [18,21,25]. It was obvious that the MetS action checklist played an important role for traditional retail market workers to create plans by themselves and promoted successful improvement outcomes in managing metabolic risk factors and lifestyle habits [12,15,25]. Simply by conducting follow-up visits to check and encourage traditional retail market workers to implement their actions for improvement, traditional retail market workers developed long-term self-reliance for changing their health behavior. This shows the cost-effectiveness of an action checklist when used in occupational health training [18,19,20,21]. The present study found that action checklists were sustainably effective in promoting self-managed MetS-related risk among traditional retail market workers.

While our subjects made efforts to achieve general goals as expressed in their action plans, our program was also efficacious in improving FBS and TGL levels among traditional retail market workers. Their FBS and TGL levels decreased starting in the second year, and more substantial reduction was noted in the third year after the intervention. This indicates that spontaneous self-management actions based on the content of the WBSM program led to reductions in FBS and TGL levels. Although there have been many reports on the results of specified intervention programs designed to give detailed advice about achieving lifestyle habits for workers [20,22,26,27], the WBSM program targeting metabolic parameters was effective for traditional retail market workers, as shown by the reductions in FBS and TGL levels, and follow-up visits encouraged traditional retail market workers to implement actions for improvements, leading to a sustainable effect.

In this study, the MetS score significantly decreased and remained in the normal range during the 3-year study period. However, HDL-C did not show any significant change and remained in the normal range during the 3-year period. Since no long-term studies have dealt with individual-level lifestyle interventions for traditional retail market workers, the finding of this study that the WBSM program on metabolic parameters produced beneficial effects on MetS score, especially on FBS and TGL, is noteworthy.

Although WC significantly decreased from baseline in the second year, it became slightly higher and reached the previous level in the third year. This might have been due to the characteristics of our study subjects; in particular, the fact that most were women, with an average age of 60.15 ± 8.51 years. It is known that the prevalence of MetS is high in Korean female workers and in those aged 60 years or more [6]. According to a study of WC and cardiometabolic risk factors [28], there were no changes in waist circumference over 3 years. Nevertheless, a 3 cm WC change is known to have beneficial effects on MetS in women [28]; therefore, it is necessary for nurses to provide a WBSM program on metabolic parameters to traditional retail market workers in the second year and intervene with more in-depth counseling to increase physical activity, modify working schedules, or arrange the environment to provide easier access to exercise facilities for female traditional retail market workers [26]. A previous study reported that both waist and BMI changes were related to changes in systolic blood pressure and hypertension [28]. However, this study showed that SBP significantly increased in the second and third years compared with the baseline but was within the normal range. This indicates that a longer intervention of the WBSM program would be needed to obtain significant decreases in blood pressure levels.

Traditional retail market workers generally have a low socioeconomic status and poor health conditions compared to other workers. There have recently been changes in intervention programs to drive workers to take the initiative in workplaces, relying on voluntary self-help actions for developing practical ideas to obtain immediate improvements [18]. Nevertheless, these were one-time interventions applied in groups [21,22,25]. The WBSM program targeting metabolic parameters for traditional retail market workers is an individual level that helps individuals self-assess their health problems. It can stimulate sustainable improvement actions for practical changes.

## 5. Conclusions

Participation in the WBSM program targeting metabolic parameters, which involved providing a MetS action checklist, counseling, creating improvement action plans, and evaluating the implemented improvements, resulted in significant improvements in FBS and TGL among traditional retail market workers. Of particular note, there were yearly reductions in FBS and TGL from the baseline during the 3 years, eventually leading to a decreased MetS score that was maintained in the normal range during the 3-year study period. The MetS action checklist allowed participants to self-assess and cope with their health problems, improve their intentions for health behavior actions, and eventually decrease their MetS score. Our study confirmed that a WBSM program targeting metabolic parameters should be considered for the primary prevention of MetS among traditional retail market workers. Future WBSM programs targeting metabolic parameters in other small business workers are needed.

The current study has some limitations. First, the study subjects were from a geographically limited area. Therefore, the findings of this study may not be generalizable to other areas of Korea or beyond. Second, all participants were sufficiently interested and motivated for behavior change to commit to participation in this study. Therefore, self-selection bias might have been present. Third, although we collected information on compliance with the action plan for 3 years, we did not fully collect data in the main areas of the MetS action checklist. Fourth, we did not establish a control group for comparison in order to evaluate the effectiveness of the WBSM program.

## Figures and Tables

**Table 1:** Action checklist for the management of metabolic syndrome.

1. Hypertension management
1-1. Self-measure and record blood pressure three times a weak
1-2. Get out of bed slowly and try to relax before going to work
1-3. Use a cart for heavy lifting and avoid maintaining the same working posture for a long time
1-4. Avoid long-distance driving to prevent blood pressure increase
1-5. Quit smoking and reduce alcohol drinking to prevent blood pressure increase
1-6. Avoid overeating and heavy drinking to maintain a normal weight and waist circumference
1-7. Maintain sleeping time more than 6 h per day
1-8. Decrease physical and mental stress by stretching and bathing frequently to lower blood pressure
1-9. Drink more than 1.5 L of water per day to prevent an elevated blood concentration
1-10. Obtain regular checks for lipid and sugar blood levels and ophthalmic examinations
2. Dyslipidemia management
2-1. Know and maintain lipid levels in the normal range
2-2. Cut fat off and eat only lean meat
2-3. Avoid eating fried food and oily soup
2-4. Reduce intake of processed products, convenient food, and junk food
2-5. Eat food with unsaturated fatty acids and fiber (nuts, fish filled with omega-3, fruits, vegetables)
2-6. Exercise for at least 30 min, three times a week to lower LDL cholesterol
3. Diabetes management
3-1. Monitor and maintain glucose levels in the normal range
3-2. Check and record glucose level at least three times a week
3-3. Eat a diet appropriate for diabetes
3-4. Exercise for at least 30 min, three times a week to maintain glucose levels
3-5. Undergo an ophthalmic examination and renal function test annually
4. Alcohol drinking management
4-1. Avoid keeping any liquor at home or at store
4-2. Make your intentions known that you are not willing to drink alcohol
4-3. Do not drink alcohol two days in a row or past your limit
4-4. Drink after eating; do not drink alcohol on an empty stomach
4-5. Drink less and more slowly by filling glasses half-full
4-6. Drink water before, during, and after consuming alcohol to prevent dehydration
4-7. Drink alcohol in a good mood; do not turn to alcohol after a stressful event
4-8. Eat vegetables, low-sodium, and protein-rich foods; avoid fried, oily, salted, and spicy food while consuming alcohol
5. Smoking habits management
5-1. Make people know that you have quit smoking; try not to meet people for dinner as doing so can break the nonsmoking habit
5-2. Throw away your cigarettes, cigarette lighters, and ashtrays
5-3. Make a list of your personal reasons for quitting and display it prominently
5-4. Wear a quit smoking badge so that everyone knows your intentions
5-5. Motivate each other to quit smoking
5-6. Practice refusal skills in case peers offer you a cigarette
5-7. Use sugar-free gum or candy, breath mints, nuts etc., as a substitute for cigarettes
5-8. Brush teeth and perform aerobic exercise right after eating a meal to resist the temptation to smoke
5-9. Engage in a hobby or activity that you enjoy so that you can deal with stress
5-10. Write out a list of good symptoms after quitting and display it prominently
6. Exercise management
6-1. Avoid prolonged postures and stretch every hour to improve impeded blood flow and stasis in veins
6-2. Stretching should be carried out more often to get into the habit of doing it
6-3. Sit and walk with a straight-spine posture to improve blood flow and burn calories
6-4. Add movement to your daily life to improve blood flow and excrete metabolic waste
6-5. Engage in at least 30 min of aerobic exercise and weight training three times a week to improve blood flow
6-6. Perform light regular exercise every day, including dumbbells, yoga, Pilates, etc.
6-7. Make people know that you are dieting and weigh yourself regularly
7. Diet management
7-1. Eat your vegetables first and avoid spicy and salted food
7-2. Eat regular breakfast slowly and with less food
7-3. Eat more fresh food and eat less processed, instant, fried, or salted food
7-4. Avoid adding extra salt into food and try not to eat salty stews
7-5. Eat fresh fruits and vegetables, take vitamin supplements, and avoid high-calorie food with large amounts of animal fats and oils
7-6. Brush one’s teeth after a meal and take an after-meal walk
7-7. Drink more than 1.5 L of water per day and avoid coffee, soda, and drinks added with artificial flavors
7-8. Eat brown or multigrain rice to slow down digestion and metabolism
8. Stress management
8-1. Identify moments when you are under stress and improve your ability to cope
8-2. Actively engage in a conversation and socializing with other merchants
8-3. Have conversations with those with a relaxed and positive mind
8-4. Have a big laugh for more than 10 s, three times a week
8-5. Relax your body and psychological tension for a while every day.
8-6. Release psychological tension by trying a life transition method or walking
8-7. Release body tension by taking a bath, having a deep sleep, and drinking enough water
8-8. Find ways to relieve your stress such as playing a sport, developing a hobby, or leading a religious life that can help you deal with your stress better

**Table 2:** Characteristics of the subjects (*n* = 70).

Variables		*n* (%), Mean ± SD
Gender	Male	21 (30.2)
	Female	49 (69.8)
Age	40–49	12 (17.1)
	50–59	28 (40.0)
	≥60	30 (42.9)
		60.15 ± 8.51
Working time (hr)	<10	3 (5.1)
	≥10	67 (94.9)
smoking	yes	23 (32.5)
	no	47 (67.5)
Drinking alcohol (days/week)	None	47 (67.1)
	1–2	15 (21.4)
	≥3	8 (11.4)
Stress	Yes	61 (87.2)
	No	9 (12.8)
Occupation-related musculoskeletal pain	Yes	38 (54.2)
	No	32 (45.8)

**Table 3:** Proposed and implemented action plans for 3 years (*n* = 70).

Variables	1st Year	2nd Year	3rd Year
	*n* (%)	*n* (%)	*n* (%)
Proposed action plans	296	281	246
Implemented action plans after 3 months	245 (82.7)	244 (86.8)	190 (77.2)
Implemented action plans after 6 months	182 (61.4)	162 (57.6)	131 (53.2)

**Table 4:** Differences in MetS risk components from baseline to follow-up in 2014, 2015, and 2016 (*n* = 70).

MetS Components	B	2014	2015	2016	B–2014	B–2015	B–2016	2014–2015	2014–2016	2015–2016	F/χ^2^	*p*
	M ± SD	M ± SD	M ± SD	M ± SD	*p*	*p*	*p*	*p*	*p*	*p*		
WC (cm)	82.97 ± 8.20	82.67 ± 8.31	81.15 ± 8.76	83.17 ± 7.64	0.617	0.009	0.753	0.016	0.402	0.006	4.44	0.005
SBP (mmHg) *	125.33 ± 14.69	126.84 ± 16.47	128.91 ± 15.69	128.52 ± 13.96	0.375	0.022	0.038	0.418	0.408	0.937	4.90	0.179
DBP (mmHg) *	76.78 ± 7.69	77.64 ± 10.20	79.01 ± 9.67	77.00 ± 8.70	0.475	0.083	0.713	0.347	0.517	0.120	3.40	0.334
FBS (mg/dL) *	100.11 ± 24.03	99.70 ± 19.88	95.02 ± 18.14	94.75 ± 19.36	0.600	0.033	0.005	0.006	<0.001	0.380	18.52	<0.001
TGL (mg/dL) *	167.98 ± 114.33	167.90 ± 126.77	150.31 ± 128.57	150.15 ± 191.22	0.452	0.021	0.009	0.074	0.008	0.468	11.68	0.009
HDL-C(mg/dL) *	47.22 ± 10.82	48.32 ± 12.70	46.92 ± 11.13	47.18 ± 11.68	0.230	0.617	0.858	0.072	0.136	0.622	4.39	0.222
Score of MetS *	2.12 ± 1.41	2.07 ± 1.31	2.02 ± 1.28	1.50 ± 1.11	0.645	0.541	<0.001	0.856	0.001	0.001	17.73	<0.001

* Wilcoxon and Friedman test was carried out due to abnormal distribution. MetS = Metabolic Syndrome; B = Baseline; WC = Waist Circumference; SBP = Systolic Blood Pressure; DBP = Diastolic Blood Pressure; FBS = Fasting Blood Sugar; TGL = Triglycerides; HDL-C = High Density Lipoprotein-Cholesterol.

## Data Availability

Not applicable.
